# Serum antibody profiling identifies vaccine-induced correlates of protection against aerosolized ricin toxin in rhesus macaques

**DOI:** 10.1038/s41541-022-00582-x

**Published:** 2022-12-16

**Authors:** Chad J. Roy, Dylan Ehrbar, Greta Van Slyke, Jennifer Doering, Peter J. Didier, Lara Doyle-Meyers, Oreola Donini, Ellen S. Vitetta, Nicholas J. Mantis

**Affiliations:** 1 Tulane National Primate Research Center, Covington, LA 70433 USA; 2 Division of Infectious Diseases, Wadsworth Center, New York State Department of Health, Albany, NY 12208 USA; 3 Soligenix, Inc, Princeton, NJ USA; 4 Departments of Immunology and Microbiology, University of Texas Southwestern Medical Center, Dallas, TX 75390 USA

**Keywords:** Protein vaccines, Adaptive immunity

## Abstract

Inhalation of the biothreat agent, ricin toxin (RT), provokes a localized inflammatory response associated with pulmonary congestion, edema, neutrophil infiltration, and severe acute respiratory distress. The extreme toxicity of RT is the result of the toxin’s B chain (RTB) promoting rapid uptake into alveolar macrophages and lung epithelial cells, coupled with the A chain’s (RTA) potent ribosome-inactivating properties. We previously reported that intramuscular vaccination of rhesus macaques with a lyophilized, alum-adsorbed recombinant RTA subunit vaccine (RiVax®) was sufficient to confer protection against a lethal dose of aerosolized RT. That study implicated RT-specific serum IgG, toxin-neutralizing activity (TNA), and epitope-specific responses as being associated with immunity. However, it was not possible to define actual correlates of protection (COP) because all vaccinated animals survived the RT challenge. We addressed the issue of COP in the current study, by vaccinating groups of rhesus macaques with RiVax® following the previously determined protective regimen (100 µg on study days 0, 30 and 60) or one of two anticipated suboptimal regimens (100 µg on study days 30 and 60; 35 µg on study days 0, 30, and 60). Two unvaccinated animals served as controls. The animals were challenged with ~5 × LD_50s_ of aerosolized RT on study day 110. We report that all vaccinated animals seroconverted prior to RT challenge, with the majority also having measurable TNA, although neither antibody levels nor TNA reached statistical significance with regard to a correlation with protection. By contrast, survival correlated with pre-challenge, epitope-specific serum IgG levels, derived from a competitive sandwich ELISA using a panel of toxin-neutralizing monoclonal antibodies directed against distinct epitopes on RiVax®. The identification of a species-neutral, competitive ELISA that correlates with vaccine-induced protection against RT in nonhuman represents an important advance in the development of medical countermeasures (MCM) against a persistent biothreat.

## Introduction

The use of biological toxins and infectious agents remains a threat to both military personnel and civilians^1–3^. Among the most concerning is ricin toxin (RT), a Type II ribosome-inactivating protein (RIP) from the castor bean plant (*Ricinus communis*)^4^. The pathophysiology associated with inhalation of RT is reminiscent of acute respiratory distress syndrome (ARDS) brought about by other biological agents, including SARS CoV-2 and bacterial exotoxins^5–8^. RT-induced lung pathology includes tissue damage, hemorrhage, inflammatory exudate, and edema^5,7^. Within hours after animals are exposed to aerosolized RT, there is a precipitous decline in alveolar macrophages (AM), disruption of the alveolar-capillary barrier, and an influx of polymorphonuclear cells (PMN) into the airways^9–12^. Disease severity and lung permeability coincide with elevated levels of pro-inflammatory cytokines such as IL-6, IL-1, and tumor necrosis factor alpha (TNF-α) in sera and bronchoalveolar lavages (BAL)^10,13–15^. Airway damage may be further exacerbated by TNF-α and its family members, which render lung epithelial cells hyper-sensitive to the effects of RT^16–18^.

In mice and nonhuman primates (NHPs), parenteral vaccination with the recombinant RT vaccine, RiVax®, protected animals against the lethal effects of aerosolized RT and had significantly less toxin-induced lung inflammation^19,20^. RiVax® consists of a lyophilized recombinant, non-toxic mutant of RT’s enzymatic subunit, RTA, adsorbed to aluminum hydroxide (alum)^19,21,22^. In rhesus macaques, three intramuscular administrations of RiVax® (100 µg) at monthly intervals conferred protection against ~3 × LD_50_ RT by aerosol^19^. IgG anti-RTA and toxin-neutralizing activity (TNA) were detected in the sera of all vaccinated animals prior to RT challenge, implicating serum antibodies as playing a central role in immunity to RT. Moreover, antibody profiling by competition ELISA with a panel of RT-neutralizing monoclonal antibodies (mAbs) suggested an “immune signature” associated with protection. Since that original report, we have refined the epitope profiling immuno-competition capture assay (EPICC). In mouse models of parenteral RT exposure following RiVax® vaccination across a range of doses, pre-challenge total RT-specific and EPICC values each correlated with survival^23–26^. However, it is not known to what degree vaccine-induced RT-specific IgG or EPICC values correlate with protection in the rhesus macaque model. Determining these relationships is essential for the development of medical MCM against biothreats like RT that will rely on the FDA’s animal rule for licensure^19^.

Therefore, in this study, we sought to investigate total RT-specific serum IgG, TNA, and EPICC as putative correlates of vaccine-induced protection in the rhesus macaque model of aerosolized RT challenge. As a strategy to stratify vaccine-induced antibody responses, we vaccinated groups of rhesus macaques with either a previously established protective dose of RiVax®^19^ or one of two anticipated suboptimal regimens of RiVax®. Serum samples were collected just prior to RT challenge and used as the basis for establishing specific marker(s) that correlate with survival.

## Results

### Experimental design and vaccination regimens

As detailed in Table 1, a total of 18 adult rhesus macaques (2 female, 16 males) were divided into four cohorts: (A) a negative control group (*n* = 2; females) that was sham (saline) vaccinated, (B) a control group (*n* = 4) that received 100 µg RiVax® intramuscularly on study days 0, 30, and 60, as done previously^19^, and two (C, D) experimental groups. Group C (*n* = 6) received 100 µg RiVax® on study days 30 and 60, while Group D (*n* = 6) received 35 µg RiVax® on days 0, 30, and 60. Group C was designed to evaluate the need for a second booster (2 × 100 µg versus the 3 × 100 µg dose regimen) in immunity to RT, while Group D received a reduced dose across the entire vaccination regimen (days 0, 30, and 60), as compared to Group B. Venous blood was collected on study days 28, 56, and 110. BAL fluids were collected on study days −7 and 111 (24 h post RT challenge)^27^. Animals were challenged with aerosolized RT on day 110^19^.

**Table 1:** RiVax vaccination groups and outcome following RT challenge.

	Animal info				Day 110		
Group^*a*^	ID^*b*^	Sex	kg^*c*^	RT^*d*^	RT IgG^*e*^	TNA_1:10_ [CI]^*f*^	Survival
**(A) control**	**GM16**	F	11.2	25.0	0	<1	-
	**GL37**	F	8.3	40.5	0	<1	-
Mean			9.75	32.75	0	<1	0/2
**(B) 3** **×** **100**	KB69	M	10.0	28.3	7.2	104	+
	JI50	M	14.1	24.4	8.8	98	+
	HG70	M	10.9	31.3	3.1	92	+
	KC20	M	11.8	31.8	6.2	89	+
Mean			11.7	29.95	6.37	95 [85–106]	4/4
**(C) 2** **×** **100**	**JK68**	**M**	**12.5**	**28.0**	**1.39**	**11**	**-**
	**KN31**	**M**	**13.0**	**31.0**	**0.6**	**<1**	**-**
	JR89	M	11.5	32.8	5.71	24	+
	JK73	M	14.4	34.7	0.79	2	+
	JN52	M	13.2	31.8	4.64	81	+
	KD85	M	11.7	29.3	0.62	<1	+
mean			12.7	31.26	2.29	20 [12–53]	4/6
**(D) 3** **×** **35**	**JR22**	**M**	**10.7**	**33.7**	**0.39**	**87**	**-**
	KI72	M	14.0	33.8	0.65	10	+
	IL74	M	14.3	21.2	0.88	94	+
	IT46	M	13.1	30.4	1.06	77	+
	KD72	M	13.2	27.5	0.76	75	+
	LB71	M	8.6	30.8	1.83	105	+
Mean			13.15	29.56	0.93	74 [39–110]	5/6

^*a*^Group designation with number and doses (mg) of RiVax; ^*b*^animal ID; ^*c*^animal weight at time of RT challenge; ^*d*^estimated RT dose in µg/kg. The average dose across groups was 29.7 ± 7.4 µg/kg or 5.1 ± 1.2 LD50; ^*e*^RT-specific serum IgG (μg/ml); ^*f*^TNA (%) at a fixed 1:10 dilution of serum.

**Fig. 1:**
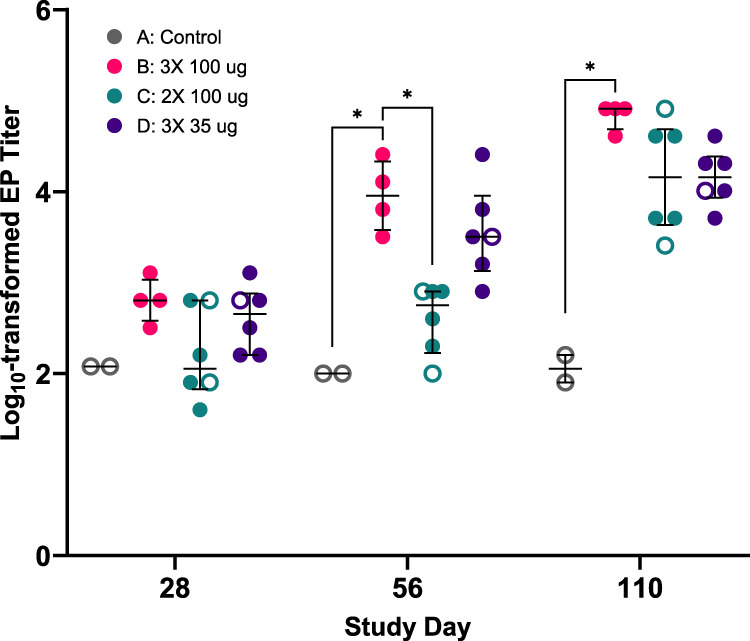
Kinetics of RT-specific endpoint titers following vaccination. Vaccinated macaques and control animals had serum samples taken on study days 28, 56, and 110, allowing for the assessment of the development of RT-specific endpoint titers over time. Dot plots are shown with central dashed lines representing the groups’ median values, and the dotted lines representing the 1st and 3rd quartiles. Statistical significance between the experimental groups is shown with asterisks (*p < 0.05, **p < 0.01), as determined by Kruskal-Wallis tests followed by Dunn’s multiple comparisons tests. Filled circles represent survivors while open circles represents decedents.

In group B (3 × 100 µg RiVax®), RT-specific antibody titers were significantly above background on day 56 (mean = 3.957 [95% CI = 3.338–4.575] log_10_-transformed reciprocal endpoint titers) and by day 110 the group’s RT-specific antibody levels had increased ~10-fold (4.838 [95% CI = 4.598–5.077]) (Table 1: Fig. 1). Group C (100 µg on days 30 and 60) had RT-specific titers of 2.602 [95% CI = 2.202–3.002] on day 56 (representing ~4 weeks after prime) and 4.161 [95% CI = 3.505–4.816] by day 110. In Group D (35 µg on days 0, 30, and 60), RT-specific IgG was significantly above background by day 56 and by day 110 had reciprocal endpoint titers of 4.161 [95% CI = 3.829–4.492]. Thus, just prior to RT challenge, Group B had slightly higher RT-specific endpoint titers compared to Groups C and D.

We assessed TNA in serum samples collected just prior to RT challenge (day 110). While TNA was detectable in serum samples from most vaccinated animals, the levels were generally too low to establish IC_50_ values in the Vero cell cytotoxicity assay. For that reason, TNA is reported as the percentage of viable cells measured 48 h after treatment with RT or RT mixed with a 1:10 dilution of serum (TNA_1:10_). By that parameter, the mean TNA_1:10_ value for Group B was 95% [95% CI = 85.32–106.13], compared to 20% [95% CI = 12–53] and 74% [95% CI = 39.26–110.04] for Groups C and D, respectively (Table 1). Assessment of RT-specific IgG and TNA in BAL fluids was inconclusive due to low signal-to-noise ratios, even when samples from vaccinated animals were normalized to naïve controls.

The absence of detectable RT-specific IgG in the BAL fluids was not surprising considering that serum IgG transudation into airways is relatively low under steady state conditions and the possibility of mechanical dilution resulting from lavage sampling techniques. In previous study, we reported that amounts of an RT-specific mAb, huPB10, in BAL fluids of rhesus macaques were only a fraction (~1/400–1000) of those in serum^14^. For example, an animal with ~200 μg/ml of huPB10 in serum had ~0.3 μg/ml in BAL fluids. In our current study, optimally vaccinated macaques (Group B) had ~6 μg/ml of RT-specific serum IgG and would therefore be expected to have <0.02 μg/ml RT-specific IgG in BAL fluids (Table 1). Even lower amounts would be present in animals in groups C and D, which likely accounts for our inability to reliability detect RT-specific IgG in BAL fluids. In recent SARS-CoV-2 vaccine studies, groups have reported concentrating BAL fluids prior to use for antibody detection, as well as reporting output values as AUC, rather than concentrations of specific IgG^28,29^.

### RT challenge, survival, and inflammatory responses

On study day 110, the control and RiVax® -vaccinated cohorts of rhesus macaques were subjected to RT challenge (~5 × LD_50_) by small particle aerosol. The two control animals (GM16, GL37) succumbed to RT intoxication within 30 h and were euthanized per protocol (Table 1). As expected, all four of the rhesus macaques in Group B (3 × 100 µg RiVax®) survived RT challenge^19^. In Group C (2 × 100 µg), four of six animals survived RT challenge, while five of six animals in Group D (3 × 35 µg) survived. In all cases, death was the result of euthanasia at the point at which animals were deemed by the lead veterinarian and the principal investigator to have reached IACUC-approved clinical criteria that warranted intervention. By this measure, there were no statistically significant differences in the number of survivors or mean time to death among the groups of RiVax® -vaccinated animals.

We employed a 29-plex Luminex array to measure pro-inflammatory cytokines, chemokines, and growth factors in serum and BAL fluids as a proxy for relative degrees of morbidity associated with RT challenge. For several of the analytes (e.g., EGF, eotaxin, FGF-basic, IFN-γ, IL-1Ra, and IL-15), we observed correlations between RiVax® dosing and RT-induced changes in analyte levels, consistent with vaccination directly impacting the inflammatory response to RT. To investigate this further, we compared analyte levels in sera collected from animals before and 24 h after RT exposure. In the two control (sham vaccinated) animals, 13 of the 29 analytes were significantly different pre- versus post-RT challenge, as determined by two-way ANOVA and subsequent Šidák’s tests (Supplementary Tables 1, 2). For example, IL-6 levels were elevated 143-fold, IL-1Ra 33-fold, MCP-1 and VEGF by 10-fold each, and IL-8 by 8-fold.

By comparison, inflammatory markers were markedly lower in sera collected from vaccinated animals after RT challenge. In Group B (3 × 100 µg), IL-6 was elevated 10-fold and IL-1Ra 6-fold, whereas in Group C (2 × 100 µg), IL-6 was elevated 27-fold, and IL-1Ra 7-fold. In Group D (3 × 35 µg), IL-6 and IL-1Ra were elevated 12- and 4-fold, respectively. These results are consistent with RiVax® -vaccinated animals having a dampened systemic inflammatory response to RT as compared to the unvaccinated control animals (which lacked RT-specific antibodies).

The same analyses were performed on the BAL fluids. In the control animals, 10 of the 29 analytes were elevated following RT challenge, as compared to pre-challenge values. The most pronounced change was in IL-6, which increased 260-fold. The following were also significantly elevated: FGF-Basic, GM-CSF, IL-1b, IL-2, IL-8, MIP-1α, MIP-1β, and TNF-α (Supplementary Tables 3, 4). Those same analytes were much less elevated or even unchanged in RiVax® -vaccinated animals (Supplementary Tables 3, 4), consistent with protection by vaccine-induced antibodies and possibly other immune effectors having a role in suppressing RT-induced local inflammation. This observation is reminiscent of a recent report from Waickman and colleagues who noted that mRNA-1273 vaccination suppressed SARS-CoV-2-induced lung inflammation^30^.

**Fig. 2:**
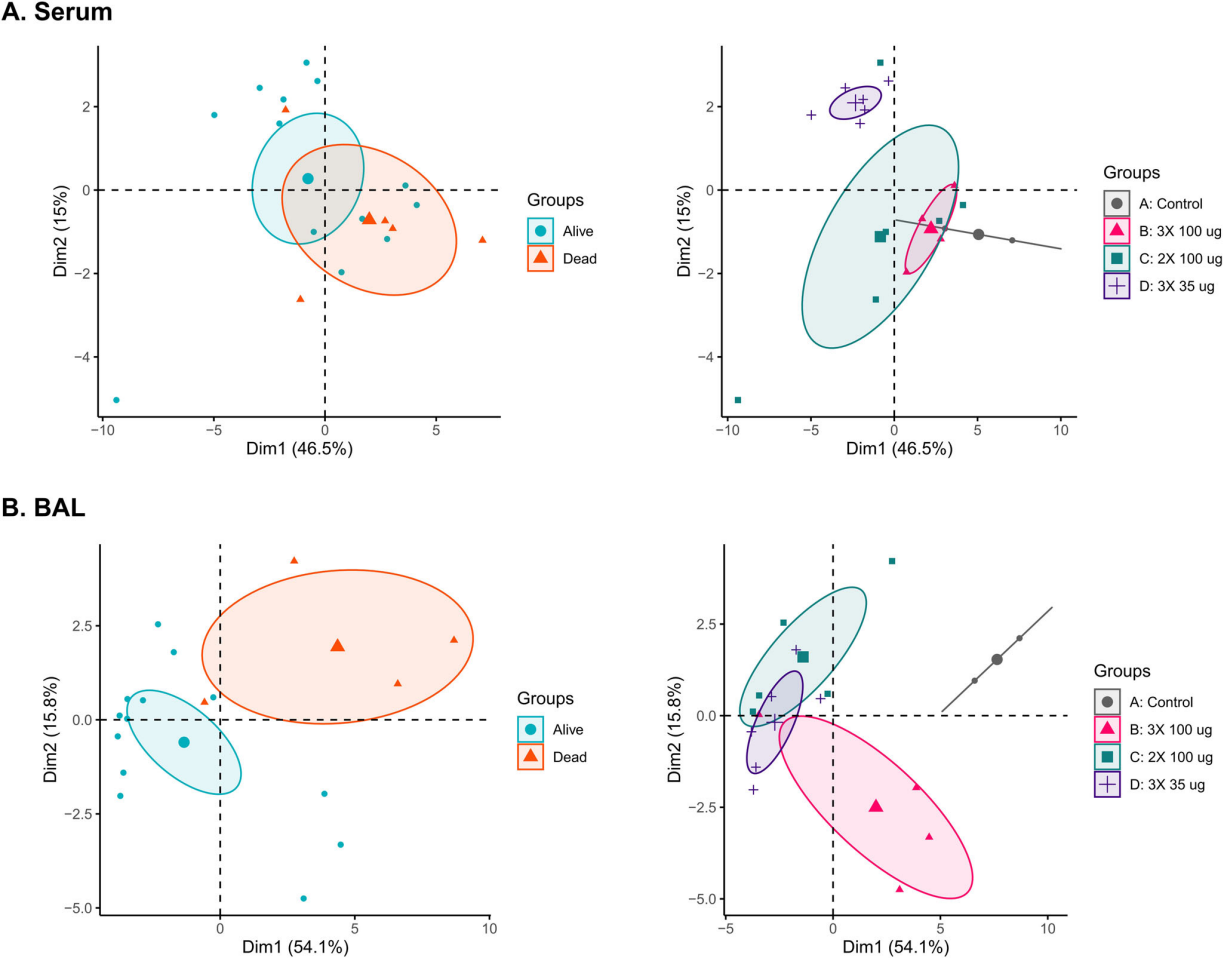
Principal component analysis (PCA) of cytokine and chemokine levels in sera and BALs. Scatterplots of principal components 1 and 2 of log_2_-transformed 29-plex analyte changes in **A** sera and **B** BALs in RT-challenged macaques. Each point represents an individual animal, with ellipses representing 95% confidence intervals around the means for each group. The left plots show ellipses around survivors and decedents, while the right plots show ellipses around each experimental group, with colors as described in the legends.

We next performed principal component analysis (PCA) to determine whether systemic (serum) or local (BAL fluids) 29-Plex profiles could discriminate between animals that ultimately survived RT challenge and those that did not. PCA based on serum 29-Plex profiles did not distinguish survivors from decedents, as evidenced by substantial overlap between the 95% confidence ellipses around the means of each group. Nor did PCA separate the four groups of RT-challenged animals, although Group D did form its own cluster possibly due to the differential levels of several serum cytokines like IL-15 (Fig. 2A; Supplementary Tables 1, 2). Analysis of BAL fluids collected 24 h post RT challenge proved more informative, as the survivors (*n* = 13) and decedents (*n* = 5) segregated into two distinct clusters, with no overlap in 95% confidence intervals around their means. PCA of BAL fluid-derived samples also differentiated the vaccinated from unvaccinated animals to a limited extent. Within the three groups of vaccinated animals, group B (3 × 100 μg) overlapped with group D (3 × 35 μg), while group D also overlapped with group C (2 × 100 μg) (Fig. 2B). The unvaccinated control animals constituted their own cluster. These results demonstrate that “local” inflammatory profiles, as revealed in the BAL fluids, are more closely associated with survival and vaccination status, than corresponding serum profiles. In other words, local suppression of RT-induced lung pathophysiology by vaccine-induced antibodies and/or other immune effector molecules reflects whether an animal survives or succumbs to RT exposure. This observation is reminiscent of recent studies in rhesus macaques regarding vaccine-induced suppression of SARS-CoV-2 lung inflammation^30^.

To identify putative signatures associated with vaccinated groups of animals, we subjected post-challenge serum- and BAL fluid-derived 29-plex data sets to hierarchical clustering (HC). HC is a machine-learning algorithm that segregates individuals from a common root based on similarity of a given dataset. Using this approach, serum analysis segregated the group of unvaccinated control animals (GM16, GL37) from the three vaccinated groups of animals. Within the vaccinated groups of animals, Group B (3 × 100 μg; red) and D (3 × 35 μg; blue) formed distinct clusters, while the six animals in Group C (2 × 100 μg; green) were interspersed in Group B (*n* = 3), Group C (*n* = 1) and as a solitary pair (*n* = 2).

**Fig. 3:**
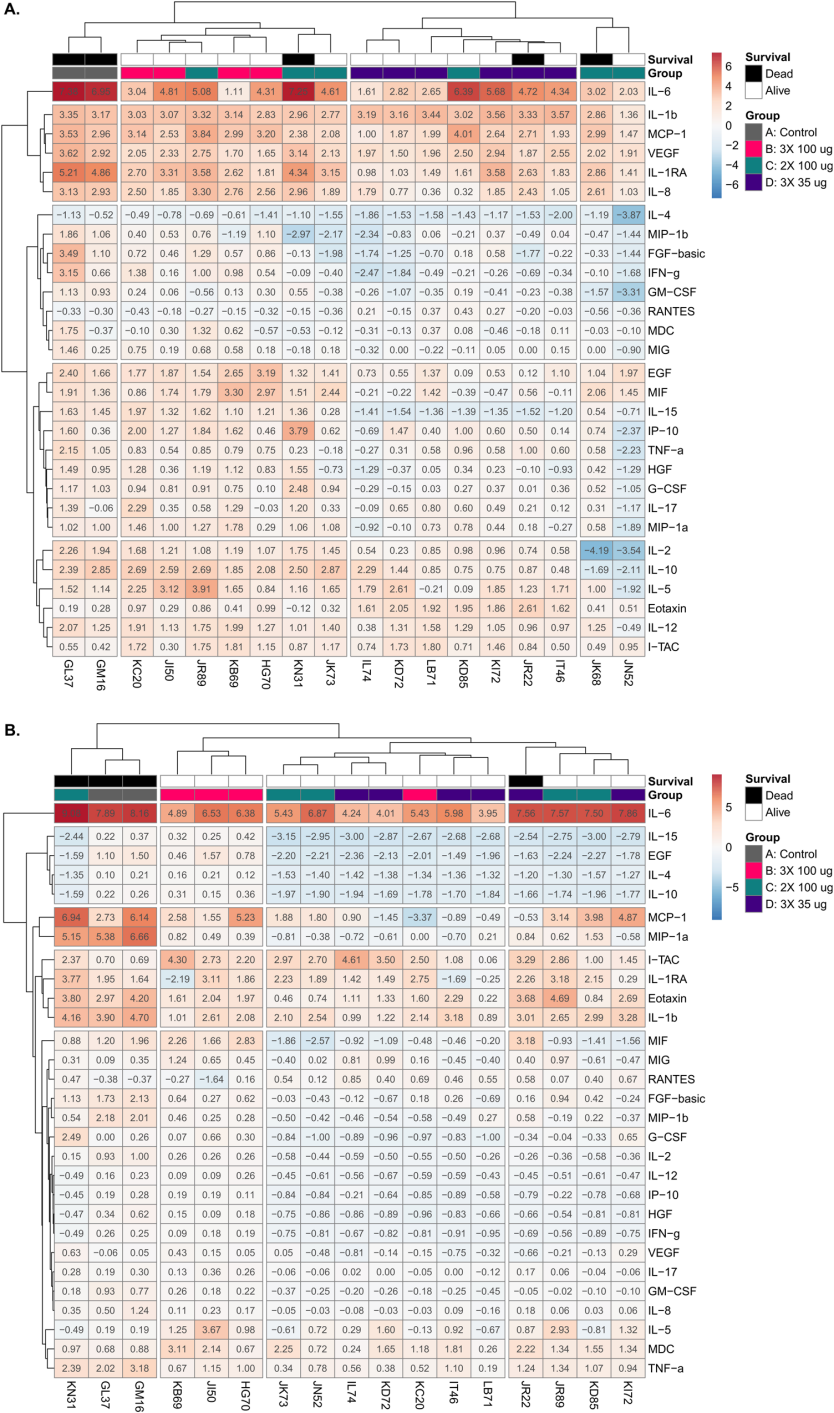
Inflammatory cytokine responses in sera and BAL fluids of control and RiVax-vaccinated Rhesus macaques following RT exposure. Heatmap (log_2_-transformed fold change) of 29-plex of cytokines, chemokines, and growth factors in **A** serum and **B** BAL fluids collected before and 24 h after RT challenge for each animal. Each column represents an animal, with the topmost row showing decedents in black and survivors in white. The next row depicts vaccine group membership (A–D), as designated in the legend. The dendrograms on the top and left of each plot show the results of unsupervised HC, which grouped of fold changes animals and cytokines with the most similar changes in expression. Each cell is scaled from red to blue, as described by the bar on the right.

IL-6 was invariably the analyte that increased the most following RT exposure (Fig. 3A). Whereas HC of serum samples was able to sort animals based on treatment group, HC of BAL fluids segregated animals based on survival. Three of the four decedents fell within a distinct cluster, with the fourth decedent (JR22; Group D) sandwiched between the survivors from Groups C and D. Once again, the analyte with the greatest fold change in BAL fluids following RT exposure was IL-6 (Fig. 3B). We conclude that local biomarkers in the lung are associated with experimental outcome following RT challenge.

### Correlates of vaccine-induced protection against RT

**Fig. 4:**
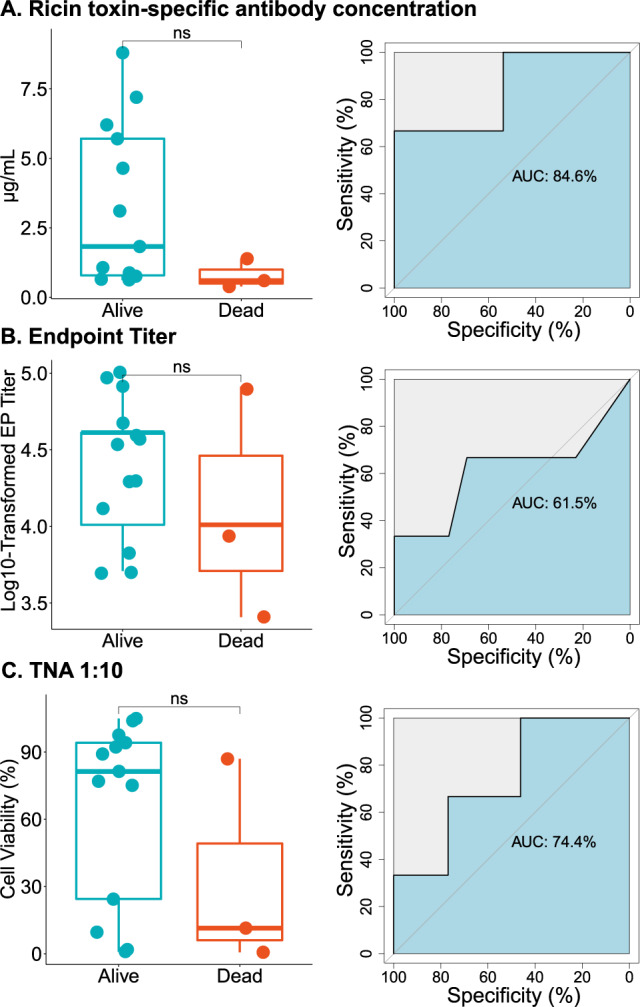
Endpoint titer and toxin-neutralizing activity as potential correlates of protection against aerosolized RT exposure. Sera were collected from control and RiVax® -vaccinated Rhesus macaques on study day 110 and then examined for **A** RT-specific antibody concentrations, **B** RT-specific endpoint titer, and **C** TNA _1:10_, as described in the “Methods”. Left panels (**A**–**C**): Levels of each analyte were compared between survivors and decedents with Mann–Whitey U tests (*p < 0.05), and results are shown in boxplots. Each point represents an individual macaque. The thick horizontal line in each box represents the median. The box defines the 1 and 3 quartiles. The whiskers extend no more than 1.5 x interquartile range, with points beyond constituting outliers. Right panels (**A**–**C**): ROC curve analysis was performed to assess the predictive ability of each analyte, with specificity plotted against sensitivity. AUC values are shown for each curve in the center of the plots, with an AUC of 100% representing a perfect predictive analyte.

We next investigated immune correlates of vaccine-induced protection against aerosolized RT exposure. In the case of inhalational anthrax, COP includes both levels of anti-protective antigen (PA) antibodies prior to challenge, and TNA (at least at some timepoints)^31^. To investigate RT-specific IgG and TNA as a COP, we compared median levels of each variable between survivors and decedents across all groups of vaccinated macaques (Groups B–D), excluding the two control animals (group A). This analysis revealed that there was a trend, but not a statistically significant difference in either RT-specific IgG [*p* = 0.08] or TNA_1:10_ [*p* = 0.2] between survivors and decedents (Fig. 4A–C; Supplementary Table 4; Supplementary Fig. 4). This contrasts with results in mice, where we have reported that RT-specific serum IgG levels strongly associated with protection against lethal dose RT challenge by injection. The lack of congruency between primate and murine results may simply be a function of group size rather than biological response. We also performed receiver operating characteristic (ROC) curve analysis to assess the predictive values of EPT and TNA^32^. ROC curve analysis is used to gauge the classification performances at predicting a given outcome, which in this case is survival following RT challenge. In the ROC curves, sensitivity (true positive rate) was plotted against specificity (true negative rate), resulting in a derived area under the curve (AUC) value for each classifying variable. AUC values provide a simple metric by which to judge the performance of each classifier and has been used in SARS-CoV-2 vaccine studies in NHPs^28,29,33,34^. In our studies, the AUC value for EPT was 76.9%, while TNA_1:10_ performed comparatively better with an AUC value of 84.6% (Fig. 4C). Thus, both EPT and TNA_1:10_ have moderate value in predicting outcome following RT challenge in the NHP model.

We next investigated whether EPICC analysis was a possible COP. EPICC is a modified sandwich ELISA in which serum samples from experimental animals are assessed for their ability to inhibit the capture of soluble, biotinylated RT by a panel of immobilized mAbs directed against immunodominant toxin-neutralizing epitopes on RiVax®^23–26^. We recently reported that EPICC inhibition values constitute a vaccine-induced COP in a mouse model of systemic RT challenge (reproduced in Table 2)^25^.

**Table 2:** Correlates of vaccine-induced immune protection to RT in NHPs, excluding naïve controls.

	Rhesus macaques^*a*^			Mice^*b*^		
	Mann–Whitney		ROC curve analysis	Mann–Whitney		ROC curve analysis
Variable	*U*	*p*-value	AUC (95% CI)	*U*	*p*-value	AUC (95% CI)
EPT	15	0.5832	61.54 (9.251–100)	72.5	0.003	78.42 (64.35–92.49)
RT IgG	6	0.0821	84.62 (53.03–100)	-^***c***^	-	-
TNA_1:10_	10	0.2393	74.36 (40.27–100)	-	-	-
R70/PB10	4	**0.0393**	89.74 (72.21–100)	65	0.002	80.65 (67.29–94.02)
SyH7	2	**0.0143**	94.87 (82.73–100)	97	0.04	71.13 (52.97–89.29)
IB2	4	**0.0393**	89.74 (72.21–100)	120	0.16	64.29 (44.82–83.75)
GD12	6	0.0821	84.62 (62.23–100)	151	0.63	55.06 (36.48–73.6)

^*a*^RT challenge by aerosol; ^*b*^RT challenge by intraperitoneal injection. Values reproduced from Van Slyke, 2020; ^*c*^dash (−) indicates analyses was not conducted, as noted in text.

The bold text highlights *p*-values < 0.05.

For the sake of this study, we used a panel of mAbs against four spatially distinct epitope clusters (I–IV) on RTA^19,25,35^. PB10 and R70 target cluster I, a solvent exposed, loop-helix-loop motif that spans RTA residues 91–116^35,36^. R70 and PB10 recognize nearly identical epitopes and were used interchangeably in this study depending on mAb availability^36^. Cluster II is located on the back face of RTA (relative to the active site) and is recognized by mAb, SyH7^35,37^. The two remaining mAbs, IB2 and GD12, recognize epitopes near RTA’s active site (IB2; cluster III) and the interface of RTA-RTB (GD12; cluster IV), respectively^35^.

**Fig. 5:**
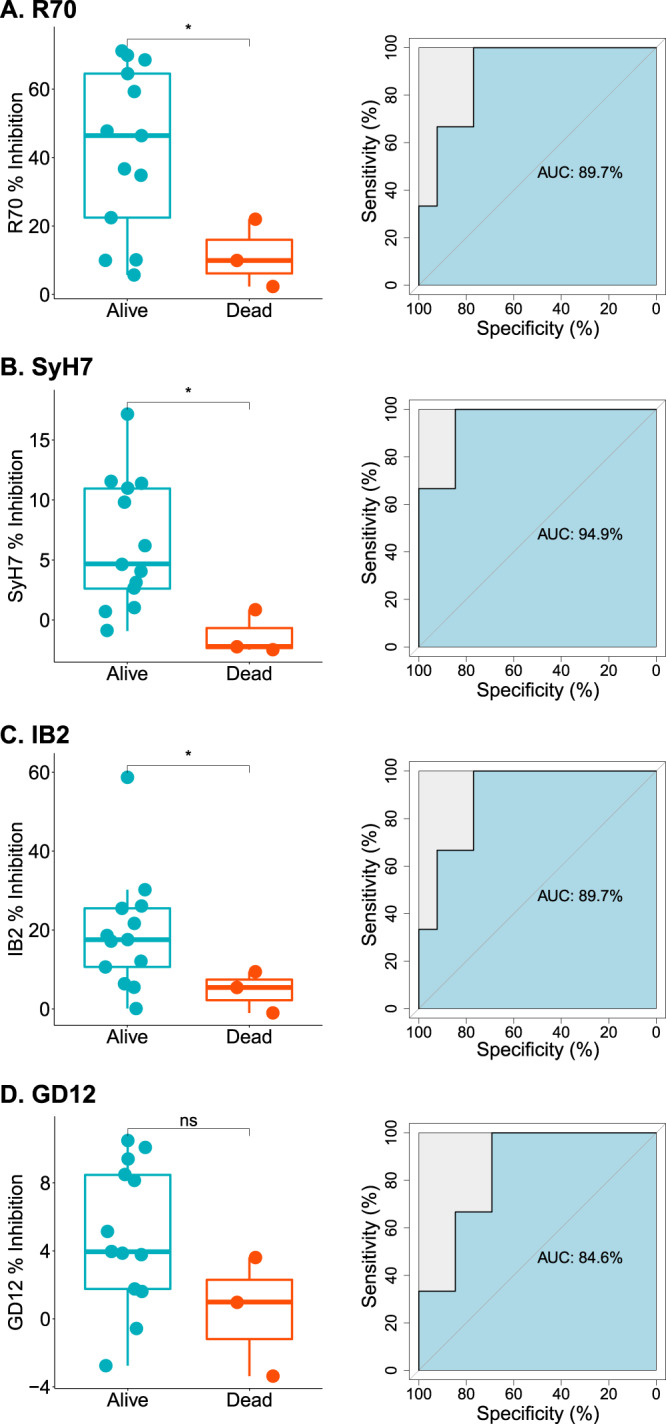
Correlations between serum IgG EPICC inhibition values and immunity to RT challenge. Serum samples from control (group A) and RiVax® -vaccinated Rhesus macaques (groups B–D) were collected on study day 110, as described in Table 1. Sample were examined for inhibitory activity in the EPICC assay using mAbs **A** R70/PB10, **B** SyH7, **C**, IB2, and **D** GD12, as described in the “Methods”. Left panels: Comparisons of EPICC inhibition values (%) between survivors and decedents are shown in boxplots on the left with asterisks denoting significant differences, as determined by Mann–Whitney U tests. The thick horizontal line in each box represents the median. The box defines the 1 and 3 quartiles, and the whiskers are max/min values. Right panels: ROC curve analysis was performed to assess the predictive ability of each analyte, with specificity plotted against sensitivity. AUC values are shown for each curve in the center of the plots, with an AUC of 100% representing a perfect predictive analyte.

EPICC analysis was performed on sera from both the control and vaccinated groups of animals collected over the course of the study (Supplementary Fig. 1). R70/PB10 inhibition values were evident in all three groups of vaccinated animals on days 56 and 110. Group B had the highest levels of R70/PB10 inhibition just prior to challenge (day 110 mean = 43% [95% CI = 12–75], followed by Group D (day 110 mean = 38% [95% CI = 12–64]), and Group C (day 110 mean = 29% [95% CI = −2–61%]). IB2 inhibition values largely mirrored those of R70/PB10, with detectable activity in all three groups of RiVax® -vaccinated animals on day 110 (B mean = 27% [95% CI = −8–63%]; D mean = 16% (95% CI = 7.0–25%), C mean = 9.5% [95% CI = −1.3–20%]). SyH7 levels were relatively low overall on days 56 and 110 (ranging from 0 to 22%). GD12 inhibition levels remained near baseline even on day 110, suggesting that cluster III epitopes are subdominant in the rhesus macaque model. EPICC inhibition values for R70/PB10, SyH7, and IB2 (but not GD12) were statistically different between vaccinated animals that survived RT exposure and those that did not (Table 2; Fig. 5). ROC analysis indicated that SyH7 afforded the most sensitivity and specificity with an AUC value of 94%, compared to 89% each for R70 and IB2. EPICC values only weakly correlated with total RT-specific IgG, as a Pearson’s correlation plot between the two parameters revealed an *R*^2^ value of 0.375 for R70 (Supplementary Fig. 2). Thus, epitope-specific antibody titers are not necessarily proportional to total RT-specific antibody titers in any given animal, further suggesting that antibody “quality” is more informative than “quantity” when predicting outcome upon RT challenge. Finally, when the EPICC protocol was modified by changing serum starting dilutions (1:50 vs. 1:25) and employing an EC_50_ value rather than EC_90_ value, the R70 and SyH7 inhibition values still correlated with survival as indicated by AUC values of 86% and 89%, respectively (Supplementary Fig. 3).

Based on these results, we propose that EPICC may serve as both a convenient and powerful method to predict vaccine-induced immunity to aerosol RT exposure in the NHP model.

## Discussion

The development and licensure of vaccines for biothreat agents for which human efficacy trials are neither ethical nor practical will depend on the use of established COP in relevant animal models of human disease. In the case of RT, the rhesus macaque is the most well-characterized NHP model of RT-induced pathophysiology and inflammation^7,13,14,19,38,39^. In this study, we found that RiVax® vaccination resulted in dose- and time-dependent onset of RT- and epitope-specific serum IgG with the original dosing regimen (3 × 100 µg at monthly intervals) being superior to the two other regimens (2 × 100 µg and 3 × 35 µg). Indeed, considering that deviations in dose or time resulted in reduced survival and elevated levels of local inflammatory responses upon RT exposure indicates that the 3 × 100 µg RiVax® regimen is a near optimal schedule in this model. Taking our previous study^19^ into account, a total of 25 rhesus macaques have survived lethal dose aerosolized RT challenge following 3 × 100 µg RiVax® dosing schedule. Unfortunately, the experimental animals in the current study were not implanted with wireless telemetry devices to enable continuous measurements of physiological functions following toxin exposure, as done in other studies^7,19^. Nonetheless, when considering survival alone, the current study when combined with our previous study constitutes an important benchmark for future RT prophylactic investigations.

While the importance of antibodies in conferring immunity to RT has been recognized for decades^14,19,40,41^, our study is the first to establish pre-challenge, serological correlates of protection in a NHP model of aerosol challenge. Indeed, in the adult rhesus macaque model, we found that RTA-specific IgG, TNA, and epitope-specific antibody levels as determined by EPICC, were each elevated in RiVax® vaccinated animals, as compared to unvaccinated controls. However, only EPICC inhibition values proved to be statistically different between vaccinated animals that survived RT challenge and those that did not. The fact that neither antibody levels nor TNA was statistically predictive of survival may be a reflection of small sample sizes (i.e., 3 decedents versus 13 survivors) rather than relevance to overall immunity to RT. In mice, RTA-specific endpoint titers and EPICC (but not TNA) correlated with protection in a model of subcutaneous RiVax® vaccination and intraperitoneal RT challenge^24–26^. In mice, combining endpoint titers and EPICC values (specifically for PB10 and SyH7) using a multivariate model resulted in higher predictive power than either one of the independent variables alone^25^. While the results of our current study suggest that employing RT-specific IgG or TNA_1:10_ along with EPICC may have added value the limited sample number of NHPs in the study did not allow more sophisticated statistical analyses at this time.

PB10 is particularly interesting as a putative readout of protection in the EPICC assay and may actually represent a mechanistic correlate of protection. PB10 targets a solvent exposed, immunodominant linear epitope on RTA situated in proximity to the active site^35,36^. R70 recognizes virtually the same epitope as PB10 ^36^. PB10’s epitope is preserved in both RiVax® and RV*Ec*, a truncated RTA subunit vaccine developed by the US Army that underwent a Phase I study several years ago^35,36,42^. PB10 is highly effective at neutralizing RT in vitro, although its mode of action remains incompletely defined. It does not block RT attachment to host cells but does interfere with trafficking of the toxin from the plasma membrane to the trans Golgi network (TGN)^43^. We have shown in the adult rhesus macaque model, that a single IV infusion of a humanized derivative of PB10 (huPB10) is sufficient to confer complete protection against a subsequent aerosolized RT exposure^14^. Moreover, PB10 was also able to rescue animals from the lethality of RT lethality if administered within ~4 h after toxin challenge^13^. While the minimum amount of circulating PB10 (threshold) required for protection against aerosolized RT challenge has not been established in primates, that number is estimated to be ~20 μg/ml in mice^24,25,44^. Translating this value to NHPs will require a larger number of animals than available to us for this study. Finally, a topic not addressed in the current study but worthy of future investigation is the possible benefit of mucosal vaccination in limiting the toxicity of aerosolized RT exposure^45,46^. As shown in Fig. 3, even RiVax® -vaccinated animals that survived RT challenge experienced local inflammation in their lungs, as evidenced by heightened pro-inflammatory cytokines in BAL fluids.

In summary, we have demonstrated a correlation between vaccine-induced, serum antibody profiles derived from a competitive ELISA and survival following lethal dose RT exposure in a NHP model. The competitive assay, which relies on a collection of toxin-neutralizing mAbs against four spatially distinct immunodominant epitopes on RiVax® proved robust in terms of both assay modifications and elimination of naïve controls from the dataset. The use of serological markers like competitive ELISAs rather than total antibody levels as correlates of vaccine-induced immunity may have applications to other respiratory agents, including influenza virus, RSV and SARS-CoV-2. Indeed, we recognize that serological assays, in general, may serve only as surrogates of protection, as they do not reflect the complete array of local antibody effector functions that exist within the lung environment^47,48^.

## Methods

### Ricin toxin (RT) and other chemical and biological reagents

Purified RT (RCA-II; RCA_60_), derived from *Ricinus communis*, was produced from castor beans at the University of Texas Southwestern^49^. We used the same lot of RT used in previous rhesus macaques challenge studies^13,14,19^. Biotinylated RT (biotin-RT) was custom ordered from Vector Laboratories (Burlingame, CA). Unless noted specifically, all other chemicals were obtained from the Sigma-Aldrich Company (St. Louis, MO).

### Rhesus macaque study design and approvals

The studies involving adult rhesus macaques (*Macaca mulatta*) were approved by the Institutional Animal Care and Use Committee (IACUC) at Tulane University, New Orleans, LA. Rhesus macaques were born and housed at the Tulane National Primate Research Center (Covington, LA; RRID:SCR_008167), which is US Department of Agriculture-licensed and fully accredited by the Association for Assessment and Accreditation of Laboratory Animal Care (AAALAC).

### Preparation of vaccine

Lyophilized RiVax® adsorbed to Alhydrogel (alum) was prepared by adsorbing 200 µg of antigen to 1 mg/mL alum equivalents as hydroxide in 10 mM histidine and 144 mM NaCl (pH 6.0) with 8% (wt/vol) trehalose, then lyophilized^19^. The vaccine was reconstituted with sterile water for injection (WFI) immediately prior to use.

### RT challenge

Aerosolization, dosing, and delivery of RT were performed on study Day 110 ^19^. The LD_50_ of RT, when delivered by small-particle aerosol, is 5.8 µg/kg in rhesus macaques^50^. For this study, the mean inhaled dose of RT across all animals was 29.7 ± 7.4 µg/kg or 5.1 ± 1.2 LD_50_s (Table 1). Aerosol exposure of RT was performed on one animal at a time. The anesthetized animals were not mechanically ventilated during the 10 min duration of RT aerosol exposure and rather were allowed to breathe in an unrestricted fashion. The actual RT dose per animal was calculated based on the product of aerosol concentration (µg/liter aerosol) and cumulative tidal volume derived from individual respiratory function measured by whole body plethysmography. Animals were euthanized at any time during the study based upon clinical assessment, biological and physiologic monitoring, and near continuous observation post-exposure in accordance with Tulane University’s IACUC. In the event that an animal reached assessment guidelines where euthanasia was recommended by the veterinarian, the animal was first anesthetized and then sodium pentobarbital was administered as per guidelines of the American Veterinary Medical Association’s Panel on Euthanasia. Following euthanasia, animals were subjected to complete necropsy. After gross necropsy, tissues were collected in neutral buffered zinc-formalin solution (Z-Fix Concentrate, Anatach, Battle Creek, MI). Fixed tissues were embedded in paraffin and sectioned for IHC^7^.

### RT-specific serum IgG concentrations and endpoint titer determinations

Native RT rather than RTA or RiVax® was used as coating antigen for ELISAs, since it represents the form of the RT used in the challenge studies. Briefly, Nunc Maxisorb F96 microtiter plates (ThermoScientific, Pittsburgh, PA) were coated with RT (1 µg/ml) in PBS, pH 7.4. Plates were blocked with 2% goat serum (Gibco, Grand Island, NY) and PBS plus 0.1% [v/v] Tween-20 (PBST). Sera were two-fold serially diluted into block solution, applied to wells, and incubated at room temperature (RT) for ~1 h. Horseradish peroxidase (HRP)-labeled goat anti-human IgG-specific polyclonal antibodies (SouthernBiotech, Birmingham, AL) were used as secondary reagents, and 3,3′,5,5′-tetramethylbenzidine (TMB; Kirkegaard & Perry Labs, Gaithersburg, MD) was used as colorimetric detection substrate, with 1 M phosphoric acid solution as the stop solution. Plates were read on a SpectroMax 250 spectrophotometer and analyzed with SoftmaxPro software (Molecular Devices, Sunnyvale, CA). End-point titers were defined as the last dilution above three-times the assay background measurement.

For RT-specific IgG concentrations, ELISA plates (Corning Incorporated, Corning, NY) were coated with RT (5 µg/ml) then blocked with StartingBlock (ThermoScientific). A standard curve was prepared using dilutions of affinity purified rabbit anti-RT polyclonal antibodies (RARA). HRP-conjugated AffiniPure goat anti-rabbit Ig (Jackson Immuno Research Labs, West Grove, PA) was used as a secondary reagent^19^. For the rhesus macaque serum samples, HRP-conjugated AffiniPure Goat anti-rhesus IgG (Southern Biotech) was used as a secondary reagent. TMB (ThermoFisher Scientific) was used as the colorimetric detection substrate and 2 M sulfuric acid as the stop solution. Samples were run in triplicate, and OD_450_ values were plotted against the standard curve. The mean value of the three replicates for each sample is reported.

### RT neutralization assays

For toxin-neutralization assays (TNA) RT (10 ng/ml) was mixed with serial dilutions of NHP serum (in duplicate) or medium alone (as a negative control) and applied to Vero cells seeded into opaque cell culture-treated 96-well plates^51^. The plates were incubated for 2 h at 37 °C, then washed to remove free RT, and then overlaid with fresh DMEM supplemented with 10% fetal bovine serum (FBS) and penicillin-streptomycin. The cells were incubated at 37 °C for 48 h before cell viability was assessed using CellTiter-GLO (Promega, Madison, WI) and read with a SpectraMax L luminometer (Molecular Diagnostics) equipped SoftMax Pro 7.0 software. The relative IC_50_ values were determined using nonlinear regression and the least squares method with the EC_anything_ function in GraphPad Prism version 9.0.

### Serum and BAL fluid cytokine arrays and analysis

Bronchoalveolar lavage (BAL) was obtained by intubation of an anesthetized animal wherein 80 ml of saline is instilled into the upper apical lobe of the lung and then recovered by the same method nearly immediately thereafter. Serum and BAL fluids were subjected to analysis using the Monkey Cytokine Magnetic 29-Plex Panel for the Luminex™ platform (ThermoFisher), which is specifically designed for quantifying monkey cytokines, chemokines, and growth factors in serum. Samples were analyzed at TNPRC using the FlexMap3D™ (Luminex Corporation, Austin, TX). Data sets were examined as detailed under the statistical analysis section. BAL fluids (in 10 ml PBS) were collected from the upper lobar bronchi and used without concentration^27^.

### Epitope profiling immuno-competition capture (EPICC) assay

Slight variations of the EPPIC assay^25^ were performed in two different labs to compare the robustness of the Results.

#### Method 1

Immulon 4HBX 96-well microtiter plates (Thermo Scientific) were coated with 0.1 mL of anti-RTA mAb (1 µg/ml) diluted in PBS (pH 7.4) and incubated overnight at room temperature. Wells were blocked at 4 °C with block solution [PBS containing 1% (v/v) Tween-20 and 2% (v/v) goat serum]. The concentration of biotinylated RT (biotin-RT) used in the EPICC assay was equivalent to the EC_90_ for each RT-specific mAb (range = 100–500 ng/ml for PB10/R70, SyH7, IB3, GD12). Biotin-RT at appropriate EC_90_ concentration was mixed with 1:25 dilutions of sera from naïve and vaccinated rhesus macaques and applied to the mAb-coated plates. The plates were incubated for 1 h at room temperature, washed, then overlaid with streptavidin-HRP (1 μg/ml). The plates were washed again to remove unbound streptavidin-HRP and developed using TMB according to the manufacturer’s instructions (Kirkegaard & Perry Labs, Gaithersburg, MD). Optical densities at 450 nm (OD_450_) for each well were determined using a SpectroMax 250 spectrophotometer (Molecular Devices) equipped with Softmax Pro 7.0 software. Inhibition of RT capture by a given mAb was expressed as a percent (%) reduction derived from the OD_450_ values of wells without (B) and with (C) competitor, as follows: [100-(OD_450_*C*/OD_450_*B*)*100].

#### Method 2

PB10 was used in place of R70 as the mAb targeting cluster I of RTA^36^. ELISA plates (Corning Incorporated, Corning, NY) were coated in 2.5 µg/ml anti-RTA mAb rather than 1 µg/ml, and plates were blocked with StartingBlock (ThermoFisher Scientific, Pittsburgh, PA) rather than 2% goat serum. EC_50_ biotin-RT solutions were also used in place of EC_90_ solutions, and 1:50 dilutions of serum in biotin-RT solution were assayed rather than 1:25 dilutions. Wells were washed with PBS containing 0.01% (v/v) Tween-20 rather 0.1% Tween-20. TMB (50 µl; ThermoFisher Scientific) per well was used as the developing agent.

### Statistical analysis

Statistical analyses were performed using GraphPad Prism 9.0 and R 4.0.2;^52^ the choice of specific tests used in the study are detailed in the Results section. The significance of intergroup differences in endpoint titers, EPICC inhibition levels, and toxin-neutralizing activities was determined by repeated measures Kruskal–Wallis tests followed by Dunn’s multiple comparisons tests. The same methods were used to examine the influence of RT exposure and treatment group on cytokine changes, but the resulting p-values were corrected to control false discovery rate (*Q* = 5%) using the Benjamini, Krieger, and Yekutieli method. The R package pheatmap^53^ was used to create heatmaps and perform hierarchical clustering. The R packages FactoMineR^54^ and factoextra^55^ were used for principal component analyses. Mann–Whitney U tests were used to compare levels of analytes between survivors and decedents and were performed with the R package ggpubr^56^. Analysis of ROC curves was performed on the same analytes using the R package pROC^57^. An alpha level of 0.05 was used in all cases.

### Reporting summary

Further information on research design is available in the Nature Research Reporting Summary linked to this article.

## Supplementary information

Supplemental Material

REPORTING SUMMARY

## Data Availability

All data generated or analyzed during this study are included in this published article (and its supplementary information files). All relevant data are available upon request from the authors.
