# Supplementary material for: Serum antibody profiling identifies vaccine-induced correlates of protection against aerosolized ricin toxin in rhesus macaques
Source: NPJ Vaccines. 2022 Dec 16;7:164. doi: 10.1038/s41541-022-00582-x (PMC9755799; doi:10.1038/s41541-022-00582-x)
Supplement: Supplementary file 1 — Supplemental Material [file 41541_2022_582_MOESM1_ESM.pdf]

Supplemental Information (Roy, Ehrbar et al.)

Supplemental Table 1. Two-way ANOVA for cytokine changes and ricin-intoxication in sera of macaques.

	EXPOSURE % VARIATION	EXPOSURE[1] SIGNFICANCE	TREATMENT % VARIATION	TREATMENT GROUP[1] SIGNIFICANCE	INTERACTION % VARIATION	INTERACTION[1] SIGNIFICANCE	MACAQUE % VARIATION	MACAQUE[1] SIGNIFICANCE
**EGF**	40.24	**0.0001**	23.85	0.1191	7.969	**0.028**	24.84	**0.0096**
**EOTAXIN**	21.17	**0.0001**	11.49	0.4165	14.33	**0.0118**	30.9	**0.0122**
**FGF-BASIC**	5.077	**0.0316**	16.22	0.1981	33.39	**0.0446**	23.61	0.4879
**G-CSF**	23.72	**0.0021**	9.504	0.4711	9.073	0.3281	31.3	0.4826
**GM-CSF**	0.01588	0.2089	13.78	0.4377	16.25	0.1518	39.93	0.3412
**HGF**	11.77	**0.0082**	17.35	0.2427	20.26	0.125	30	0.4620
**IFN-G**	3.711	**0.0395**	13.89	0.3433	32.66	**0.0394**	29.44	0.4349
**IL-1B**	79.71	**0.0001**	0.6594	0.4165	0.7877	0.1937	1.709	0.4349
**IL-1RA**	75.62	**0.0001**	6.308	0.1767	7.814	**0.0499**	7.934	0.4349
**IL-2**	11.66	**0.0132**	10.69	0.4711	12.06	0.2974	35.49	0.4826
**IL-4**	39.41	**0.0001**	3.554	0.6267	4.584	0.2974	19.36	0.3412
**IL-5**	25.82	**0.0005**	8.411	0.5795	2.005	0.6687	38.06	0.3100
**IL-6**	72.99	**0.0001**	6.076	0.0851	5.34	0.1518	4.996	0.6742
**IL-8**	45.31	**0.0001**	0.7123	0.9505	3.254	0.1966	44.18	**0.0046**
**IL-10**	37.18	**0.0002**	14.96	0.1981	7.359	0.3281	21.31	0.4879
**IL-12**	51.19	**0.0001**	4.359	0.7213	2.531	0.3597	30.43	0.0523
**IL-15**	4.983	**0.0083**	36.15	**0.0028**	39.99	**0.0026**	11.51	0.4879
**IL-17**	18.78	**0.0038**	1.94	0.93	4.863	0.5279	44.62	0.3412
**I-TAC**	26.83	**0.0001**	5.286	0.7639	1.75	0.4083	47.19	**0.0046**
**MCP-1**	74.44	**0.0001**	3.007	0.4165	2.375	0.1966	7.454	0.3412
**MDC**	2.749	0.0613	5.246	0.8218	4.853	0.518	60.87	0.2089
**MIF**	14.42	**0.0001**	39.46	0.0851	5.921	0.0664	34.85	**0.0046**
**MIG**	4.833	**0.0057**	2.793	0.93	6.912	0.125	79.21	**0.0046**
**MIP-1A**	19.52	**0.0036**	7.99	0.5795	10.84	0.2974	35.51	0.4349
**MIP-1B**	0.00472	0.2091	5.06	0.7639	18.43	0.1518	44.67	0.3412
**RANTES**	4.441	**0.0057**	31.58	0.2427	4.136	0.1966	52.49	**0.0046**
**TNF-A**	19.33	**0.0016**	9.292	0.6012	10.25	0.2074	45.88	0.2238
**VEGF**	76.02	**0.0001**	7.714	**0.0284**	2.313	0.0906	4.305	0.3412
**IP-10**	18.43	**0.0057**	8.049	0.5664	3.334	0.6687	32.37	0.4879

[1] FDR-corrected p-values from two-way ANOVAs. Q = 5%.

Supplemental Table 2. Šidák's tests results comparing cytokine levels pre- and post-intoxication in sera of macaques.

POST- VS PRE-EXPOSURE VALUES WITHIN GROUPS[1]

	A Mean Dif.	A p-value	B Mean Dif.	B p-value	C Mean Dif.	C p-value	D Mean Dif.	D p-value
**EGF**	2.029 (.8665 to 3.191)	**0.0008**	2.370 (1.548 to 3.192)	**<0.0001**	1.228 (0.5573 to 1.900)	**0.0005**	0.7334 (0.06222 to 1.405)	**0.0298**
**EOTAXIN**	0.2330 (-0.7911 to 1.257)	0.9498	0.6652 (-0.05889 to 1.389)	0.078	0.6575 (0.06625 to 1.249)	**0.0268**	1.945 (1.354 to 2.536)	**<0.0001**
**FGF-BASIC**	2.295 (0.2729 to 4.318)	**0.0235**	0.6537 (-0.7763 to 2.084)	0.6164	-0.4002 (-1.568 to 0.7675)	0.8154	-0.8495 (-2.017 to 0.3182)	0.2084
**G-CSF**	1.099 (-0.3603 to 2.558)	0.184	0.6493 (-0.3824 to 1.681)	0.3264	0.6782 (-0.1641 to 1.521)	0.1417	0.05624 (-0.7861 to 0.8986)	0.9995
**GM-CSF**	1.032 (-0.7108 to 2.775)	0.3813	0.1826 (-1.050 to 1.415)	0.9893	-0.8456 (-1.852 to 0.1606)	0.1182	-0.4514 (-1.458 to 0.5548)	0.6321
**HGF**	1.223 (-0.3329 to 2.778)	0.1562	0.8949 (-0.2050 to 1.995)	0.1357	0.2451 (-0.6530 to 1.143)	0.9078	-0.4033 (-1.301 to 0.4948)	0.6313
**IFN-G**	1.904 (0.04923 to 3.759)	**0.0432**	0.7667 (-0.5448 to 2.078)	0.3931	-0.2469 (-1.318 to 0.8239)	0.9474	-1.015 (-2.086 to 0.05573)	0.0665
**IL-1B**	3.256 (2.383 to 4.129)	**<0.0001**	3.017 (2.399 to 3.634)	**<0.0001**	2.715 (2.211 to 3.219)	**<0.0001**	3.375 (2.871 to 3.879)	**<0.0001**
**IL-1RA**	5.034 (3.109 to 6.960)	**<0.0001**	2.610 (1.248 to 3.972)	**0.0003**	2.825 (1.713 to 3.937)	**<0.0001**	1.922 (0.8103 to 3.034)	**0.0009**
**IL-2**	2.098 (-1.181 to 5.377)	0.3119	1.288 (-1.031 to 3.607)	0.4408	-0.4119 (-2.305 to 1.481)	0.957	0.6497 (-1.244 to 2.543)	0.8148
**IL-4**	-0.8260 (-2.308 to 0.6561)	0.4375	-0.8222 (-1.870 to 0.2259)	0.1574	-1.639 (-2.495 to -0.7834)	**0.0003**	-1.612 (-2.468 to -0.7568)	**0.0004**
**IL-5**	1.328 (-1.398 to 4.055)	0.561	1.964 (0.03630 to 3.892)	**0.0451**	0.9816 (-0.5924 to 2.556)	0.3345	1.497 (-0.07716 to 3.071)	0.0654
**IL-6**	7.163 (3.783 to 10.54)	**0.0001**	3.317 (0.9274 to 5.707)	**0.0057**	4.729 (2.778 to 6.680)	**<0.0001**	3.637 (1.686 to 5.589)	**0.0004**
**IL-8**	3.032 (1.305 to 4.758)	**0.0008**	2.421 (1.200 to 3.642)	**0.0002**	2.018 (1.022 to 3.015)	**0.0002**	1.374 (0.3777 to 2.371)	**0.006**
**IL-10**	2.621 (-0.2400 to 5.482)	0.0791	2.305 (0.2818 to 4.328)	**0.023**	0.8338 (-0.8181 to 2.486)	0.5292	1.113 (-0.5393 to 2.765)	0.2683
**IL-12**	1.660 (0.4773 to 2.844)	**0.0052**	1.577 (0.7403 to 2.414)	**0.0004**	1.037 (0.3541 to 1.720)	**0.0027**	1.041 (0.3578 to 1.724)	**0.0027**
**IL-15**	1.537 (0.07258 to 3.001)	**0.038**	1.400 (0.3642 to 2.435)	**0.0069**	0.2835 (-0.5619 to 1.129)	0.8267	-1.399 (-2.244 to -0.5536)	**0.0013**
**IL-17**	0.6691 (-0.8583 to 2.197)	0.6514	0.9732 (-0.1068 to 2.053)	0.0857	0.3102 (-0.5717 to 1.192)	0.8014	0.3618 (-0.5201 to 1.244)	0.702
**I-TAC**	0.4838 (-0.5882 to 1.556)	0.6272	1.246 (0.4881 to 2.004)	**0.0014**	0.9907 (0.3718 to 1.610)	**0.0017**	1.179 (0.5598 to 1.798)	**0.0004**
**MCP-1**	3.248 (1.778 to 4.718)	**<0.0001**	2.966 (1.926 to 4.005)	**<0.0001**	2.794 (1.946 to 3.643)	**<0.0001**	2.023 (1.175 to 2.872)	**<0.0001**
**MDC**	0.6894 (-0.5715 to 1.950)	0.4554	0.06493 (-0.8266 to 0.9565)	0.9993	0.1050 (-0.6229 to 0.8330)	0.9904	-0.1002 (-0.8282 to 0.6278)	0.9919
**MIF**	1.634 (-0.1789 to 3.447)	0.0856	2.215 (0.9334 to 3.497)	**0.0009**	1.478 (0.4314 to 2.525)	**0.0049**	0.1622 (-0.8845 to 1.209)	0.9874
**MIG**	0.8523 (0.004390 to 1.700)	**0.0486**	0.4276 (-0.1720 to 1.027)	0.2232	-0.05431 (-0.5438 to 0.4352)	0.9965	-0.05713 (-0.5467 to 0.4324)	0.9957
**MIP-1A**	1.010 (-0.6914 to 2.711)	0.3791	1.131 (-0.07251 to 2.334)	0.0696	0.4804 (-0.5019 to 1.463)	0.5577	0.009240 (-0.9731 to 0.9916)	>0.9999
**MIP-1B**	1.464 (-0.7897 to 3.719)	0.2987	0.2074 (-1.387 to 1.801)	0.9935	-1.083 (-2.385 to 0.2180)	0.1231	-0.5326 (-1.834 to 0.7689)	0.7039
**RANTES**	-0.3156 (-0.8308 to 0.1996)	0.3504	-0.2673 (-0.6316 to 0.09703)	0.2023	-0.2122 (-0.5096 to 0.08528)	0.223	0.07780 (-0.2196 to 0.3752)	0.9197
**TNF-A**	1.600 (0.01750 to 3.183)	**0.047**	0.7284 (-0.3908 to 1.848)	0.2972	0.03537 (-0.8785 to 0.9492)	>0.9999	0.4692 (-0.4446 to 1.383)	0.5136
**VEGF**	3.274 (2.335 to 4.213)	**<0.0001**	1.933 (1.269 to 2.597)	**<0.0001**	2.408 (1.866 to 2.951)	**<0.0001**	2.134 (1.592 to 2.677)	**<0.0001**
**IP-10**	0.9772 (-1.694 to 3.649)	0.7788	1.336 (-0.5528 to 3.226)	0.2294	0.9383 (-0.6043 to 2.481)	0.3569	0.4020 (-1.140 to 1.945)	0.9206

[1] p-values from Šidák's multiple comparisons tests following two-way ANOVAs.

Supplemental Table 3. Two-way ANOVA for cytokine changes and ricin-intoxication in BAL fluid of macaques.

	EXPOSURE % VARIATION	EXPOSURE[1] SIGNFICANCE	TREATMENT % VARIATION	TREATMENT GROUP[1] SIGNIFICANCE	INTERACTION % VARIATION	INTERACTION[1] SIGNIFICANCE	MACAQUE % VARIATION	MACAQUE[1] SIGNIFICANCE
**EGF**	7.186	**0.0035**	20.13	**0.0045**	35.12	**0.0004**	8.32	0.6683
**EOTAXIN**	53.87	**0.0001**	6.374	0.2659	3.424	0.2777	20.74	0.6349
**FGF-BASIC**	32.09	**0.0001**	10.21	0.1699	38.34	**0.001**	23.24	0.6349
**G-CSF**	0.3611	0.1839	24.46	0.0721	4.765	0.3281	32.85	0.6492
**GM-CSF**	9.716	**0.0018**	14.36	0.123	49.13	**0.0004**	25.52	0.6349
**HGF**	10.2	**0.0013**	15.74	**0.0381**	27.52	**0.0007**	15.26	0.6349
**IFN-G**	14.53	**0.0002**	19.71	**0.0145**	23.54	**0.0007**	12.8	0.6349
**IL-1B**	72.71	**0.0001**	7.375	0.0506	6	**0.0369**	8.277	0.6349
**IL-1RA**	24.46	**0.0008**	5.81	0.4528	7.842	0.1432	39.74	0.6349
**IL-2**	0.6887	0.1159	21.86	**0.0345**	42.35	**0.0004**	19.96	0.6349
**IL-4**	22.84	**0.0001**	19.65	**0.004**	21.38	**0.0004**	6.689	0.6349
**IL-5**	10.51	**0.0203**	16.46	0.1242	5.329	0.2999	30.9	0.6474
**IL-6**	81.47	**0.0001**	2.635	**0.0447**	1.842	0.0771	2.763	0.6524
**IL-8**	31.56	**0.0001**	21.03	**0.0182**	36.32	**0.001**	15	0.6349
**IL-10**	21.96	**0.0001**	18.5	**0.0045**	22.29	**0.0004**	7.159	0.6349
**IL-12**	12.45	**0.0004**	23.07	**0.0134**	22.87	**0.0007**	14.22	0.6349
**IL-15**	22.95	**0.0001**	19.7	**0.004**	21.3	**0.0004**	6.794	0.6349
**IL-17**	20.37	**0.0023**	8.738	0.3601	14.77	0.0771	38.73	0.6349
**I-TAC**	41.84	**0.0001**	2.728	0.4528	4.571	0.1658	18.98	0.6349
**MCP-1**	28.31	**0.0014**	4.76	0.4528	12.98	0.1205	30.02	0.6349
**MDC**	45.9	**0.0001**	2.299	0.484	2.749	0.2958	19.36	0.6349
**MIF**	3.47	0.0788	11.76	0.1774	25.93	**0.0444**	28.21	0.6537
**MIG**	7.266	**0.0348**	18.92	0.1242	5.976	0.2948	34.97	0.6349
**MIP-1A**	27.81	**0.0002**	24.78	**0.0134**	34.81	**0.001**	14.7	0.6349
**MIP-1B**	18.5	**0.0005**	16.91	0.062	45.05	**0.0004**	20.92	0.6349
**RANTES**	0.4251	0.1649	23.59	0.0724	17.09	**0.0444**	32.52	0.6349
**TNF-A**	64.5	**0.0001**	12.48	**0.0267**	14.09	**0.0101**	10.26	0.6349
**VEGF**	0.5524	0.1184	3.75	0.6125	4.782	0.123	78.82	**0.0176**
**IP-10**	11.78	**0.0023**	23.18	**0.0134**	18.95	**0.0103**	14.3	0.6349

[1] FDR-corrected p-values from two-way ANOVAs. Q = 5%.

Supplemental Table 4. Šidák's tests results comparing cytokine levels pre- and post-intoxication in BAL fluid of macaques.

	POST- VS PRE-EXPOSURE VALUES WITHIN GROUPS[1]							
	**A Mean Dif.**	**A p-value**	**B Mean Dif.**	**B p-value**	**C Mean Dif.**	**C p-value**	**D Mean Dif.**	**D p-value**
**EGF**	1.302 (-0.3084 to 2.913)	0.1376	0.2026 (-0.9363 to 1.342)	0.9783	-2.101 (-3.120 to -1.082)	**0.0002**	-1.892 (-2.822 to -0.9620)	**0.0002**
**EOTAXIN**	3.584 (0.7884 to 6.380)	**0.0106**	1.805 (-0.1719 to 3.782)	0.0798	2.105 (0.3370 to 3.873)	**0.0176**	1.887 (0.2728 to 3.501)	**0.0198**
**FGF-BASIC**	1.929 (0.9870 to 2.870)	**0.0002**	0.4285 (-0.2373 to 1.094)	0.3022	0.4046 (-0.1909 to 1.000)	0.2574	-0.2156 (-0.7593 to 0.3280)	0.7205
**G-CSF**	0.1315 (-1.793 to 2.056)	0.9994	0.01775 (-1.343 to 1.378)	>0.9999	0.05693 (-1.160 to 1.274)	0.9999	-0.5634 (-1.674 to 0.5474)	0.5185
**GM-CSF**	0.8511 (0.4892 to 1.213)	**<0.0001**	0.1202 (-0.1357 to 0.3761)	0.5867	-0.1123 (-0.3412 to 0.1165)	0.5481	-0.2193 (-0.4283 to -0.01040)	**0.0381**
**HGF**	0.4789 (-0.1194 to 1.077)	0.1433	-0.1363 (-0.5593 to 0.2868)	0.8419	-0.6844 (-1.063 to -0.3060)	**0.0007**	-0.7842 (-1.130 to -0.4387)	**<0.0001**
**IFN-G**	0.2584 (-0.2752 to 0.7920)	0.5602	-0.08781 (-0.4651 to 0.2895)	0.944	-0.6988 (-1.036 to -0.3613)	**0.0002**	-0.7986 (-1.107 to -0.4905)	**<0.0001**
**IL-1B**	4.304 (2.430 to 6.178)	**<0.0001**	1.962 (0.6364 to 3.287)	**0.0036**	2.889 (1.703 to 4.074)	**<0.0001**	2.095 (1.013 to 3.177)	**0.0004**
**IL-1RA**	1.797 (-1.341 to 4.935)	0.4067	1.382 (-0.8368 to 3.601)	0.3306	2.644 (0.6589 to 4.628)	**0.0081**	0.5851 (-1.227 to 2.397)	0.8408
**IL-2**	0.9630 (0.4080 to 1.518)	**0.001**	0.06040 (-0.3321 to 0.4529)	0.9873	-0.3642 (-0.7152 to -0.01318)	**0.0407**	-0.4146 (-0.7351 to -0.09416)	**0.01**
**IL-4**	0.1586 (-0.6003 to 0.9175)	0.9614	-0.2123 (-0.7490 to 0.3243)	0.7223	-1.430 (-1.910 to -0.9502)	**<0.0001**	-1.325 (-1.763 to -0.8865)	**<0.0001**
**IL-5**	0.1926 (-2.385 to 2.770)	0.9992	1.442 (-0.3807 to 3.265)	0.1502	0.3494 (-1.281 to 1.980)	0.9578	0.7231 (-0.7652 to 2.211)	0.5572
**IL-6**	8.023 (5.194 to 10.85)	**<0.0001**	5.807 (3.807 to 7.808)	**<0.0001**	7.289 (5.500 to 9.079)	**<0.0001**	5.600 (3.966 to 7.233)	**<0.0001**
**IL-8**	0.8693 (0.4730 to 1.266)	**0.0001**	0.1190 (-0.1612 to 0.3992)	0.6701	0.07273 (-0.1779 to 0.3234)	0.8848	0.01086 (-0.2179 to 0.2397)	0.9999
**IL-10**	0.2427 (-0.7947 to 1.280)	0.943	-0.2410 (-0.9746 to 0.4926)	0.8325	-1.829 (-2.485 to -1.173)	**<0.0001**	-1.766 (-2.365 to -1.167)	**<0.0001**
**IL-12**	0.1975 (-0.2005 to 0.5954)	0.5384	-0.03804 (-0.3194 to 0.2434)	0.9922	-0.5342 (-0.7859 to -0.2825)	**0.0001**	-0.5293 (-0.7591 to -0.2995)	**<0.0001**
**IL-15**	0.2951 (-1.222 to 1.812)	0.9701	-0.4202 (-1.493 to 0.6525)	0.7292	-2.858 (-3.817 to -1.898)	**<0.0001**	-2.760 (-3.636 to -1.884)	**<0.0001**
**IL-17**	0.2441 (-0.02977 to 0.5179)	0.0896	0.1740 (-0.01963 to 0.3676)	0.0862	0.03635 (-0.1368 to 0.2095)	0.9608	0.001917 (-0.1562 to 0.1600)	>0.9999
**I-TAC**	0.6932 (-1.848 to 3.234)	0.9053	2.932 (1.135 to 4.729)	**0.0016**	2.378 (0.7710 to 3.985)	**0.0036**	2.333 (0.8661 to 3.800)	**0.002**
**MCP-1**	4.433 (-0.9146 to 9.781)	0.1239	1.499 (-2.282 to 5.281)	0.7209	3.547 (0.1645 to 6.929)	**0.0384**	0.4016 (-2.686 to 3.489)	0.9933
**MDC**	0.7784 (-0.8553 to 2.412)	0.5744	1.775 (0.6200 to 2.930)	**0.0027**	1.368 (0.3346 to 2.401)	**0.0085**	1.252 (0.3090 to 2.195)	**0.0083**
**MIF**	1.582 (-1.414 to 4.578)	0.4816	1.567 (-0.5516 to 3.685)	0.1937	-1.178 (-3.073 to 0.7166)	0.3322	-0.1749 (-1.905 to 1.555)	0.9974
**MIG**	0.2206 (-0.9749 to 1.416)	0.9753	0.6252 (-0.2201 to 1.470)	0.1937	0.05916 (-0.6969 to 0.8152)	0.9991	0.1450 (-0.5452 to 0.8352)	0.9607
**MIP-1A**	6.021 (3.149 to 8.894)	**0.0002**	0.4239 (-1.607 to 2.455)	0.9616	1.222 (-0.5950 to 3.039)	0.2656	-0.2622 (-1.921 to 1.396)	0.986
**MIP-1B**	2.097 (1.190 to 3.005)	**<0.0001**	0.1006 (-0.5412 to 0.7424)	0.9864	-0.06800 (-0.6421 to 0.5060)	0.9953	-0.1667 (-0.6908 to 0.3573)	0.8476
**RANTES**	-0.3750 (-1.400 to 0.6495)	0.7733	-0.2675 (-0.9919 to 0.4570)	0.7681	0.3209 (-0.3271 to 0.9689)	0.5401	0.5849 (-0.006644 to 1.176)	0.0531
**TNF-A**	2.602 (1.442 to 3.762)	**<0.0001**	0.8351 (0.01504 to 1.655)	**0.0453**	1.184 (0.4506 to 1.918)	**0.0018**	0.7370 (0.06748 to 1.407)	**0.0288**
**VEGF**	-0.006001 (-0.7589 to 0.7469)	>0.9999	0.1195 (-0.4129 to 0.6519)	0.9505	-0.02847 (-0.5047 to 0.4477)	0.9997	-0.3991 (-0.8338 to 0.03563)	0.0777
**IP-10**	0.2364 (-0.4237 to 0.8965)	0.7865	-0.08895 (-0.5557 to 0.3778)	0.9722	-0.6244 (-1.042 to -0.2069)	**0.0033**	-0.6342 (-1.015 to -0.2531)	**0.0014**

[1] p-values from Šidák's multiple comparisons tests following two-way ANOVAs.

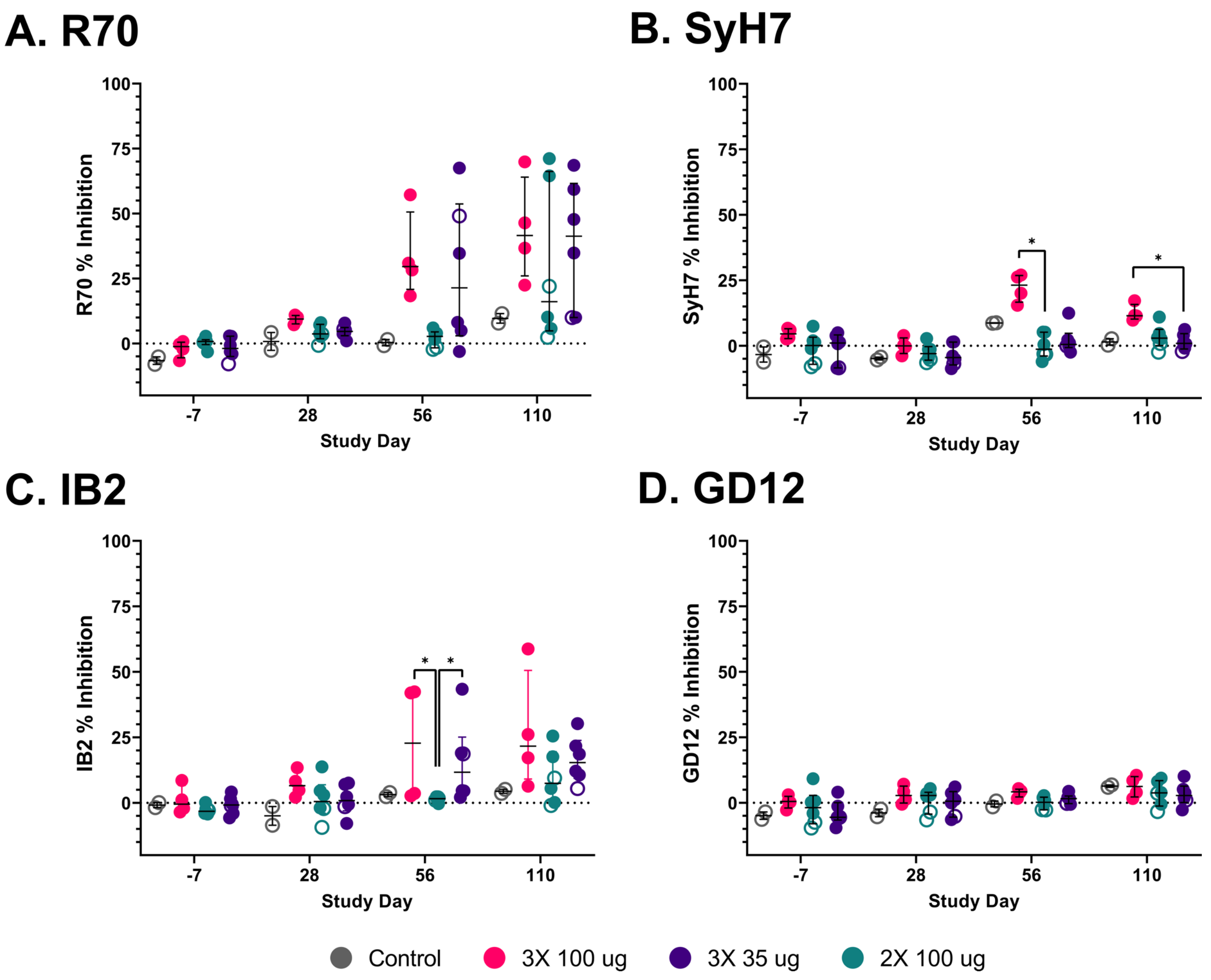

Supplemental Figure 1: Kinetics of EPICC inhibition following vaccination. Serum samples taken from rhesus macaques (control and vaccinated) on study days -7, 28, 56, and 110 were subjected EPICC using mAbs R70 (A), SyH7 (B), IB2 (C), and GD12 (D). Dot plots are shown with central dashed lines representing the groups' median values, with the dotted lines representing the 1st and 3rd quartiles. Statistical significance between the experimental groups is shown with asterisks (* $p < 0.05$, ** $p < 0.01$), as determined by Kruskal-Wallis tests followed by Dunn's multiple comparisons tests. Filled circles represent survivors, while open circles represent decedents.

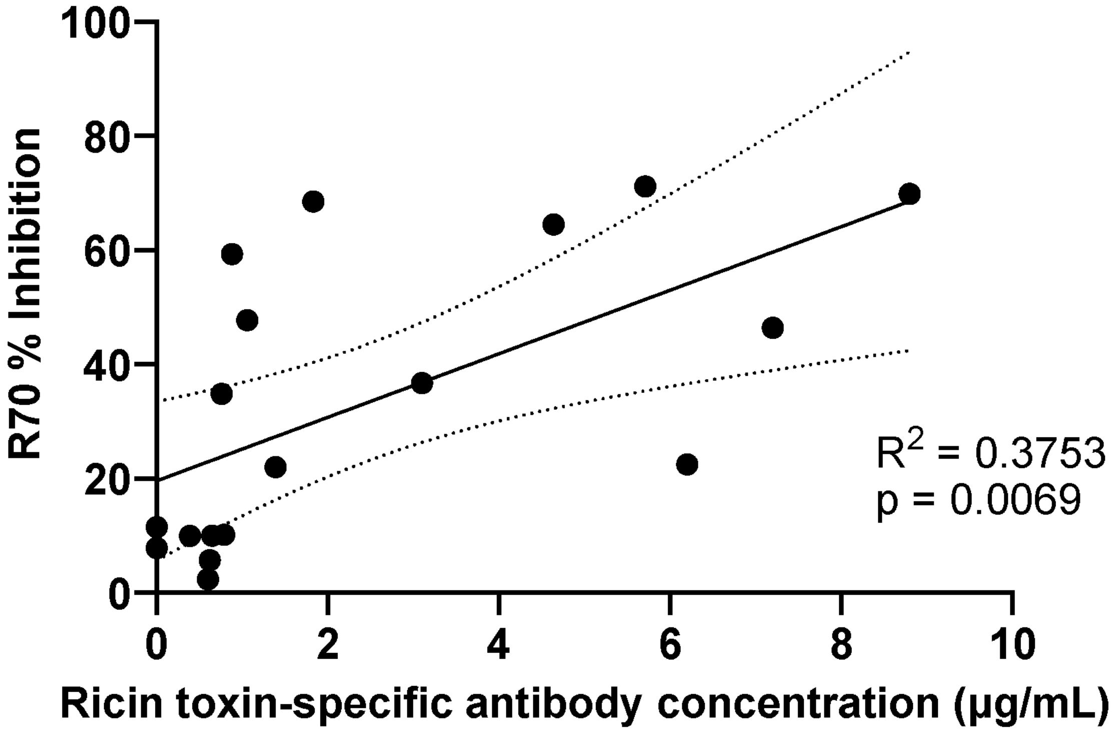

Supplemental Figure 2. Correlation analysis between total RT-specific IgG and R70 EPICC inhibition values. Shown is a Pearson's correlation plot comparing total RT-specific IgG and R70 EPICC inhibition values derived from Figures 4 and 5. While there is a correlation between the two variables (R^2 = 0.3753), the relationship is relatively weak. Furthermore, a measurement of variance inflation factor (VIF) in a logistic regression model yielded a VIF of 1.15, which is not indicative of collinearity.

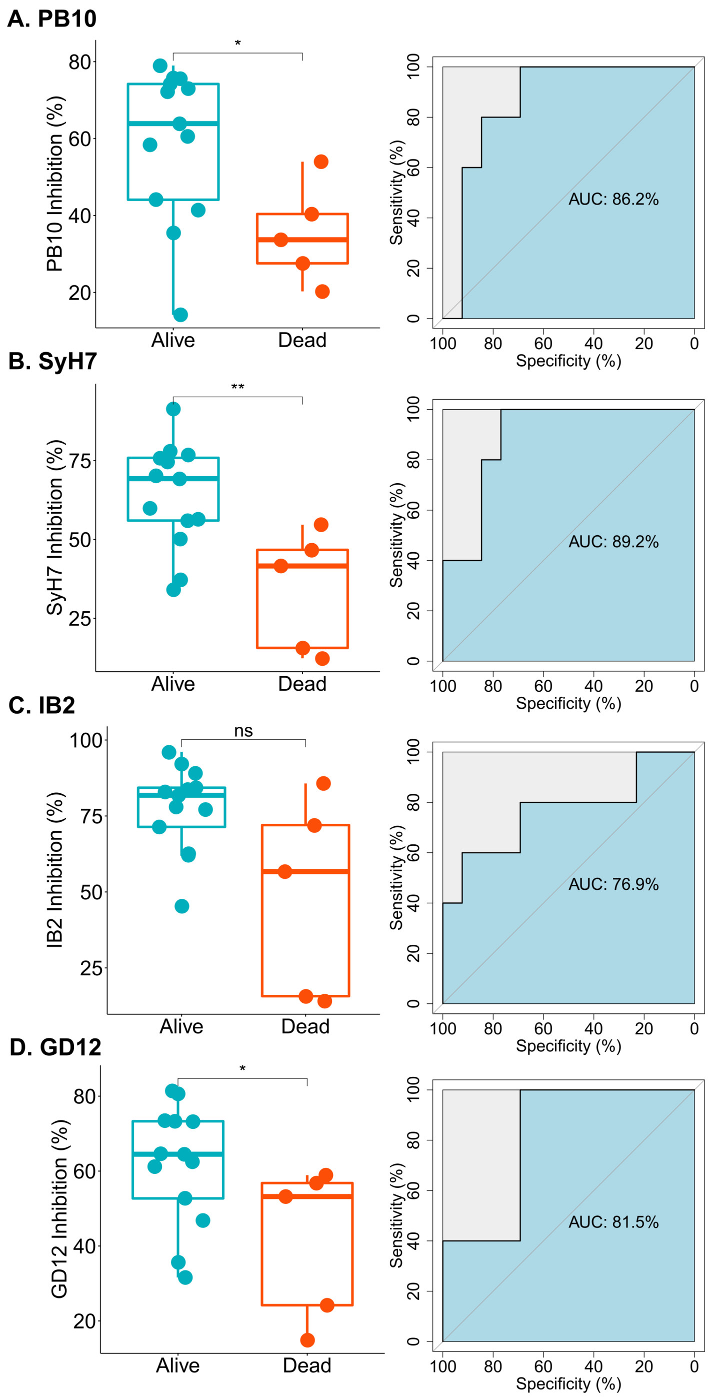

Supplemental Figure 3. Robustness of EPICC inhibition values as correlates of protection. Serum samples taken from rhesus macaques (control and vaccinated) on study days 110 were subjected EPICC using mAbs R70 (A), SyH7 (B), IB2 (C), and GD12 (D), as described Results and in Figure S1. **Left panels:** Levels of each analyte were compared between survivors and decedents with Mann-Whitey U tests (* p< 0.05), and results were shown in boxplots in the figures on the left. The thick horizontal line in each box represents the median. The box defines the 1 and 3 quartiles, and the whiskers are max/min values. Each point represents an individual macaque. **Right panels:** ROC curve analysis was performed to assess the predictive ability of each analyte, with specificity plotted against sensitivity. AUC values are shown for each curve in the center of the plots, with an AUC of 100% representing a perfect predictive analyte.
